# Fractal Analysis of Elastographic Images for Automatic Detection of Diffuse Diseases of Salivary Glands: Preliminary Results

**DOI:** 10.1155/2013/347238

**Published:** 2013-05-16

**Authors:** Alexandru Florin Badea, Monica Lupsor Platon, Maria Crisan, Carlo Cattani, Iulia Badea, Gaetano Pierro, Gianpaolo Sannino, Grigore Baciut

**Affiliations:** ^1^Department of Cranio-Maxillo-Facial Surgery, University of Medicine and Pharmacy “Iuliu Haţieganu”, Cardinal Hossu Street 37, 400 029 Cluj-Napoca, Romania; ^2^Department of Clinical Imaging, University of Medicine and Pharmacy “Iuliu Haţieganu”, Croitorilor Street 19-21, 400 162 Cluj-Napoca, Romania; ^3^Department of Histology, Pasteur 5-6 University of Medicine and Pharmacy “Iuliu Haţieganu”, 400 349 Cluj-Napoca, Romania; ^4^Department of Mathematics, University of Salerno, Via Ponte Don Melillo, 84084 Fisciano, Italy; ^5^Department of Dental Prevention, University of Medicine Pharmacy “Iuliu Haţieganu”, Victor Babes Street, 400 012 Cluj-Napoca, Romania; ^6^Department of System Biology, Phd School, University of Salerno, Via Ponte Don Melillo, 84084 Fisciano, Italy; ^7^Department of Oral Health, University of Rome Tor Vergata, Viale Oxford, 00100 Rome, Italy

## Abstract

The geometry of some medical images of tissues, obtained by elastography and ultrasonography, is characterized in terms of complexity parameters such as the fractal dimension (FD). It is well known that in any image there are very subtle details that are not easily detectable by the human eye. However, in many cases like medical imaging diagnosis, these details are very important since they might contain some hidden information about the possible existence of certain pathological lesions like tissue degeneration, inflammation, or tumors. Therefore, an automatic method of analysis could be an expedient tool for physicians to give a faultless diagnosis. The fractal analysis is of great importance in relation to a quantitative evaluation of “real-time” elastography, a procedure considered to be operator dependent in the current clinical practice. Mathematical analysis reveals significant discrepancies among normal and pathological image patterns. The main objective of our work is to demonstrate the clinical utility of this procedure on an ultrasound image corresponding to a submandibular diffuse pathology.

## 1. Introduction

In some recent papers [1–4], the fractal nature of nucleotide distribution in DNA has been investigated in order to classify and compare DNA sequences and to single out some particularities in the nucleotide distribution, sometimes in order to be used as markers for the existence of certain pathologies [5–9]. Almost all these papers are motivated by the hypothesis that changes in the fractal dimension might be taken as markers for the existence of pathologies since it is universally accepted nowadays that bioactivity and the biological systems are based on some fractal nature organization [3, 4, 10–13]. From a mathematical point of view, this could be explained by the fact that the larger the number of interacting individuals, the more complex the corresponding system of interactions is. These hidden rules that lead to this complex fractal topology could be some simple recursive rules, typical of any fractal-like structure, which usually requires a large number of recursions in order to fill the space. 

In recent years, many papers [3–6, 9, 14, 15] have investigated the multi-fractality of biological signals such as DNA and the possible influence of the fractal geometry on the functionality of DNA from a biological-chemical point of view. Almost all these papers concerning the multifractality of biological signals are based on the hypothesis that the functionality and the evolution of tissues/cells/DNA are related to and measured by the evolving fractal geometry (complexity), so that malfunctions and pathologies can be linked with the degeneracy of the geometry during its evolution time [5–7, 16–18].

From a mathematical point of view, a fractal is a geometric object mainly characterized by the noninteger dimension and self-similarity so that a typical pattern repeats itself cyclically at different scales. A more complex definition of a fractal is based on the four properties: self-similarity, fine structure, irregularities, and noninteger dimension [19]. The fractal dimension is a parameter which measures the relationship between the geometric un-smoothness of the object and its underlying metric space. Since it is a noninteger value, it is usually taken as a measure of the unsmoothness, thus being improperly related to the level of complexity or disorder. Fractality has been observed and measured in several fields of specialization in biology, similar to those in pathology and cancer models [20, 21]. However, only recently have been made some attempts to investigate the structural importance of the “fractal nature” of the DNA. It has been observed in some recent papers that the higher FD corresponds to the higher information complexity and thus to the evolution towards a pathological state [3, 4].

In the following, we will analyse the particularities of the fractal dimension focused on the pathological aspects of some tissues, more specific those belonging to a submandibular gland. For the first time, the FD is computed on images obtained by the new technology of elastographic imaging focused on this salivary gland. 

## 2. Materials and Methods

### 2.1. Material

A 55-year-old woman presented herself in the emergency room of the Maxilo-Facial Surgery Department for acute pain and enlargement of the left submandibular gland and was selected for ultrasound evaluation. The ultrasound examination was performed using the ACUSON S2000 (Siemens) ultrasound equipment, where the ARFI (acoustic radiation force impulse) and real-time elastography technique were implemented. The ACUSON S2000 is a powerful, non-invasive, ultrasound based device, which gives very accurate B mode and Doppler images of tissues. It has been profitably used for the analysis of abdominal, breast, cardiac, obstetrical, and gynaecological imaging and also for small parts such as thyroid and vascular imaging. 

The patient was placed laying down and facing up, while the transducer was placed in contact with skin on the area of the right and then the left submandibular gland successively. The shear wave velocity within the right and the left submandibular gland parenchyma was determined for each submandibular gland (in meters/second); colour elastographic images were also acquired. A colour map was used where stiff tissues were coded in blue and soft tissues in red. These images were studied afterwards for fractal analysis.

**Figure 1:**
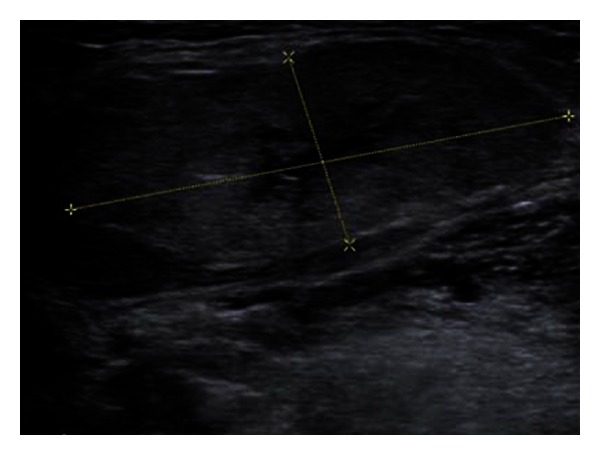
Gray scale ultrasonography of the submandibular gland (right side). The gland is enlarged (total volume around 12 cmc) with well-defined delineation, inhomogeneous structure, hypoechoic area in the center (belongs to the hilum of the gland), and hyperechoic areas under the capsule (belong to the parenchyma).

**Figure 2:**
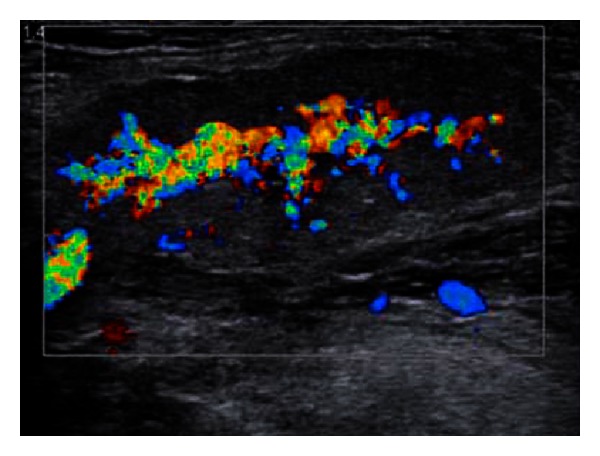
Colour coded Doppler ultrasonography (same case as Figure 1). In the central part of the gland there are vessels (blue and red according to the direction of the blood flow in relation to the transducer). The amplitude and extension of the colour signal are suggestive of hyperaemia (in this case it was an acute inflammation of the submandibular salivary gland).

**Figure 3:**
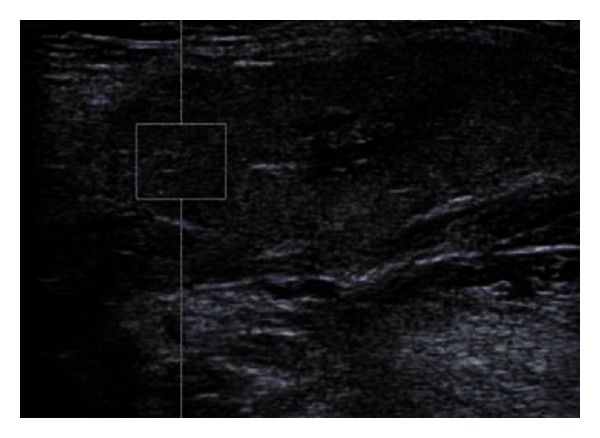
Elastogram of the submandibular gland (on the right side, inflamed gland) using the ARFI procedure. The measurements are made in an area of glandular parenchyma, in a predefined rectangular area, vessel free. The ultrasound speed is 2,55 m/sec.

**Figure 4:**
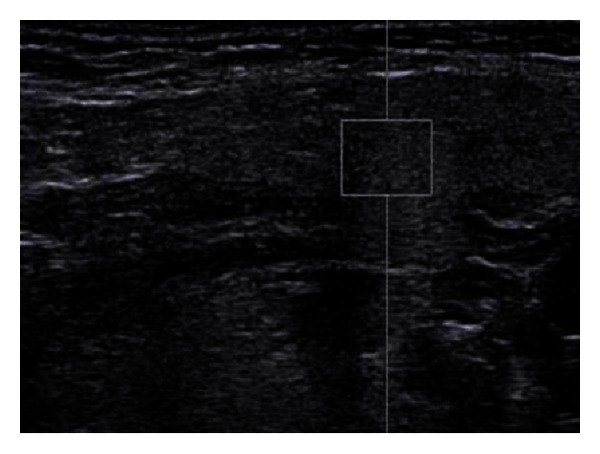
Elastogram of the submandibular gland (left side, normal gland) by means of ARFI procedure. The sample rectangle is positioned subscapular, in a similar position as it was on the right side gland. The ultrasound speed in the measured area is 1,36 m/sec.

**Figure 5:**
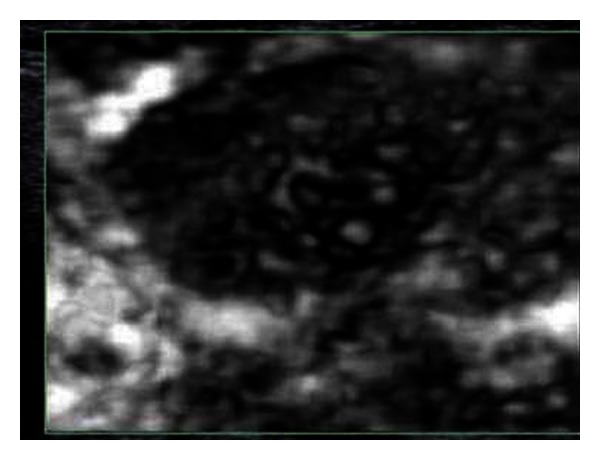
Qualitative (black and white coded; black is rigid; white is soft) elastogram (ARFI procedure) of the submandibular inflamed gland (right side). The pathological area inside the gland is well defined. This area presents a high rigidity index in relation to the amplitude of the pathological process.

**Figure 6:**
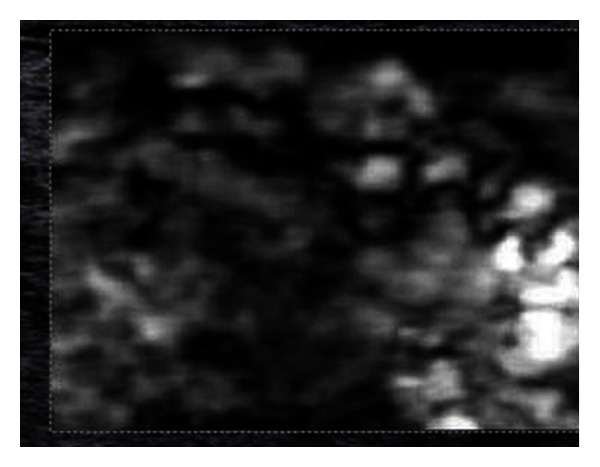
Qualitative (black and white coded; black is rigid; white is soft) elastogram (ARFI procedure) of the normal gland (considered to be the “witness,” on the left side). The dispersion of the vectors of speed is obvious. There is no obvious compact hard parenchyma as in the right pathological gland (Figure 5).

Figure 1 represents a 2D ultrasound evaluation in a “grey scale” mode, and Figure 2 represents a combination between 2D ultrasonography and “colour flow map” (CFM, or “duplex sonography”). From the first viewing, we can easily detect, by its enlargement, the gland swelling (Figure 1) and the hyper vascular pattern (Figure 2), both of these pieces of information being highly suggestive for the inflammation diagnosis. The combined clinical and ultrasound evaluation is conclusive for an acute inflammation of the submandibular gland. Figures 3 and 5 (obtained on the right salivary swollen gland) and Figures 4 and 6 (obtained on the left side, normal gland) represent elastography in quantitative mode (Figures 3 and 4), color mode (Figures 5 and 6) (ARFI tissue imaging mapping color). 

### 2.2. Methods

Concerning the fractal analysis in this section, we will summarize some definitions already given in [3].

### 2.3. Parameters for the Analysis of Complexity and Fractal Geometry

As a measure of the complexity and fractal geometry, we will consider only the fractal dimension and regression analysis (Shannon information entropy, lacunarity, and succolarity will be considered in a forthcoming paper).

Let *p*_*x*_(*n*) be the probability to find the value *x* at the position *n*, the fractal dimension is given by [3, 4, 22]

(1) $$\begin{array}{c} D=\frac{1}{N}\sum_{n=2}^{N}\frac{\log\;p_{x}\left(n\right)}{\log\;n}. \end{array}$$

In order to compute the FD, we will make use of the gliding box method on a converted black and white image. Let *S*_*N*_ be a given black and white image (BW) with 1 and 0 in correspondence with respectively, black and white pixels, we can consider a gliding box of *r*-length, so that

(2) $$\begin{array}{c} \mu_{r}\left(k\right)=\sum_{s=k}^{k+r-1}v_{\text{sh}}^{*} \end{array}$$

is the frequency of “1” within the box. The corresponding probability is

(3) $$\begin{array}{c} p_{r}\left(k\right)=\frac{1}{r}\sum_{s=k}^{k+r-1}v_{\text{sh}}^{*}. \end{array}$$

Then the box moves to the next position *k* + 1 so that we obtain the probability distribution

(4) $$\begin{array}{c} {\left\{p_{r}\left(k\right)\right\}}_{k=1,\ldots ,N}, \end{array}$$

so that we can compute the frequency of “1” within the box. The FD is computed on such gliding boxes through (1).

## 3. Results

### 3.1. Fractal Dimension for 2D Ultrasound and Elastographic Images

**Figure 7:**
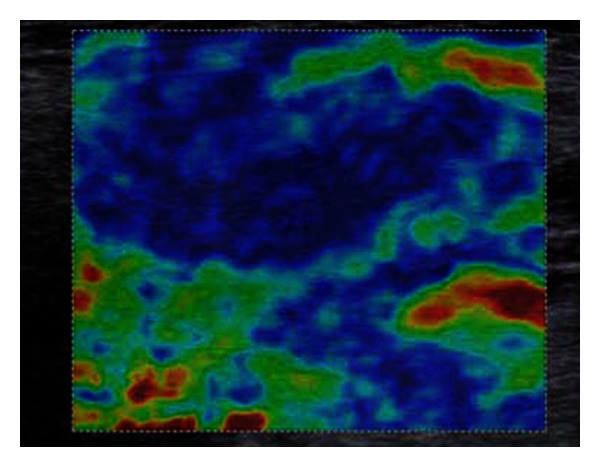
Real-time elastography (qualitative colour coded elastography; blue is rigid; red is soft) obtained by the compression of the right submandibular gland. The blue colour is in direct relation to the rigid parenchyma which is considered to be pathological.

**Figure 8:**
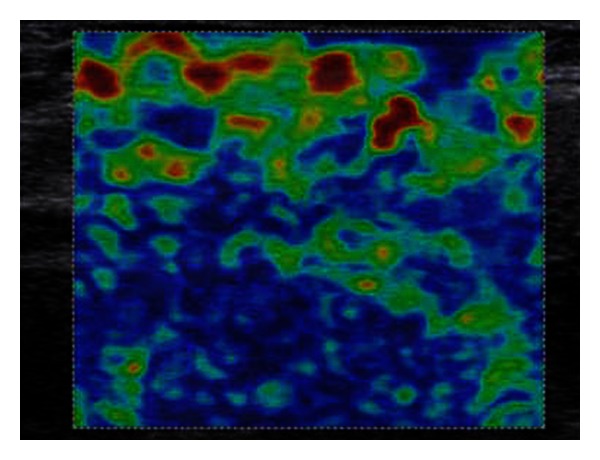
Real-time elastography (qualitative colour coded elastography; blue is rigid; red is soft) obtained by the compression of the left submandibular gland (normal). This is a normal pattern for the gland, suggestive of parts of different elasticity.

Concerning the fractal dimension of the elastographic images, as given by (1), we can see (Table 1) that the highest FD is shown by Figure 7 and lowest by the Figure 8.

The images were analyzed in 8-bit using the Image J software (tools box counting). 

The figures are referred to a patient with an acute inflammation of the submandibular gland.

Figure 1 shows a 2D ultrasound evaluation in grey scale. Figure 2 shows a 2D colour flow map evaluation (duplex sonography). Figures 3 and 4 were obtained by using the method elastography ARFI-Siemens, and they display quantitative information. The values of fractal dimension (FD) of Figures 3 and 4 are similar, and it is not possible to distinguish between pathological (Figure 3) and normal (Figure 4) states. The Figures 5 and 6 are obtained through elastography ARFI with qualitative information. From the fractal analysis by the box counting method, we have noticed that the value of Fd is lower (1.650) in Figure 5 (pathological condition) than Figure 6 (normal state). Figures 7 (pathological state) and 8 (normal state) were obtained through real time elastography.

From the computations, we can note that the higher value of Fd belongs to the pathological state (1.907), thus suggesting that the Fd increases during the evolution of the pathology (increasing degeneracy). Therefore, from Fd, analysis is possible to distinguish between pathological state and normal state of tissues by real time elastography because it is the better method to discriminate Fd values in a clear, sharp way.

## 4. Discussion

Elastography is an ultrasonographic technique which appreciates tissue stiffness either by evaluating a colour map [23, 24] or by quantifying the shear wave velocity generated by the transmission of an acoustic pressure into the parenchyma (ARFI technique) [25–27]. In the first situation, the visualization of the tissue stiffness implies a “real-time” representation of the colour mode elastographic images overlapped on the conventional gray-scale images, each value (from 1 to 255) being attached to a color. The system uses a color map (red-green-blue) in which stiff tissues are coded in dark blue, intermediate ones in shades of green, softer tissues in yellow and the softest in red, but the color scale may be reversed in relation to how the equipment is calibrated. Depending on the color and with the help of a special software, several elasticity scores that correlate with the degree of tissue stiffness can be calculated [23]. Numerous clinical applications using these procedures were introduced into routine practice, many of them being focused on the detection of tumoral tissue in breast, thyroid, and prostate.

In the last years, a new elastographic method, based on the ARFI technique (acoustic radiation force impulse imaging), is available on modern ultrasound equipment. The ARFI technique consists in a mechanical stimulation of the tissue on which it is applied by the transmission of a short time acoustic wave (<1 ms) in a region of interest, determined by the examiner, perpendicular on the direction of the pressure waves, and leading to a micronic scale “dislocation” of the tissues. Therefore, in contrast with the usual ultrasonographic examination, where the sound waves have an axial orientation, the shear waves do not interact directly with the transducer. Furthermore, the shear waves are attenuated 10.000 faster than the conventional ultrasound waves and therefore need a higher sensitivity in order to be measured [25–29]. Detection waves, which are simultaneously generated, have a much lower intensity than the pressure acoustic wave (1 : 1000). The moment when the detection waves interact with the shear waves represents the time passed from the moment the shear waves were generated until they crossed the region of interest. The shear waves are registered in different locations at various moments and thus the shear wave velocity is automatically calculated, the stiffer the organ the higher the velocity of the shear waves. Therefore, the shear wave velocity is actually considered to be an intrinsic feature of the tissue [25–29]. In current clinical practice, the same transducer is used both to generate the pressure acoustic wave and to register the tissue dislocation. Since the technique is implemented in the ultrasound equipment through software changes, B mode ultrasound examination, color Doppler interrogation and ARFI images are all possible on the same machine [30].

Currently, elastography is widely studied in relation to different clinical applications: breast, thyroid, liver, colon and prostate [29, 31–36]. The application in salivary gland pathology has been singularly considered at least in our literature database. Some reports present the utility of elastography in a better delineation of tumors of these glands. Applications on diffuse disease are few although the importance of this kind of pathology is important! Inflammations of salivary glands occur in many conditions and the incidence is significant. There is a need for accurate diagnosis, staging, and prognosis. The occurrence of complications is also very important! Elastography represents a “virtual” way of palpation reproductive and with possibility of quantification. 

Although there are several improvements, the main limitation of elastography is the dependency of the procedure to the operator's experience. This characteristic makes elastography vulnerable with a quite high amount of variations of elastographic results and interpretation. A more accurate analysis of the elastographic picture based on very precise evaluation as fractal analysis is an obvious step forward. In our preliminary study, the difference between normal and pathologic submandibular tissue using the fractal analysis was demonstrated. Because of the very new technologies accessible in practice as elastography is, and because of the mathematical instruments available as fractal analysis of the pictures, we are encouraged to believe that the ultrasound procedure might become operator independent and more confident for subtle diagnosis. However, a higher number of pictures coming from different patients with diffuse diseases in different stages of evolution are needed.

## 5. Conclusion

In this work, the multi-fractality of 2D and elastographic images of diffuse pathological states in submandibular glands has been investigated. The corresponding FD has been computed and has shown that images with the highest FD correspond to the existence of pathology. The extension of this study with incrementing the number of ultrasound images and patients is needed to demonstrate the practical utility of this procedure.

## Figures and Tables

**Table 1:** Fractal values.

Type of image	Fractal value
2D evaluation ultrasound grey scale	1.777
Duplex sonography	1.754
ARFI (quantitative)—Ps	1.771
ARFI (quantitative)—Ns	1.796
ARFI (qualitative)—Ps	1.650
ARFI (qualitative)—Ns	1.701
Real-time elastography—Ps	1.907
Real-time elastography—Ns	1.543

Ps: pathological state, Ns: normal situation.
